# Preparation of Carbon Fiber Electrodes Modified with Silver Nanoparticles by Electroplating Method

**DOI:** 10.3390/ma18133201

**Published:** 2025-07-07

**Authors:** Yuhang Wang, Rui Li, Tianyuan Hou, Zhenming Piao, Yanxin Lv, Changsheng Liu, Yi Xin

**Affiliations:** 1 Southern Marine Science and Engineering Guangdong Laboratory (Zhanjiang), Zhanjiang 524000, China; wangyh21@mails.jlu.edu.cn (Y.W.); lir22@mails.jlu.edu.cn (R.L.); houty21@mails.jlu.edu.cn (T.H.); piaozm23@mails.jlu.edu.cn (Z.P.); lv.yanxin@hotmail.com (Y.L.); 2 College of Instrumentation and Electrical Engineering, Jilin University, Changchun 130061, China

**Keywords:** silver nanoparticles, carbon fibers, nonpolarizing electrodes, electroplating, electric field sensor

## Abstract

To solve the problems of carbon fiber (CF) electrodes, including poor frequency response and large potential drift, CFs were subjected to a roughening pretreatment process combining thermal oxidation and electrochemical anodic oxidation and then modified with Ag nanoparticles (AgNPs) using electroplating to prepare a CF electric field sensor. The surface morphology of the as-prepared AgNP-CF electric field sensor was characterized via optical microscopy, scanning electron microscopy, XPS, and energy-dispersive spectroscopy, and its impedance, polarization drift, self-noise, and temperature drift values were determined. Results show that the surface modification of the AgNP-CF electric field sensor is uniform, and its specific surface area is considerably increased. The electrode potential drift, characteristic impedance, self-noise, and temperature drift are 52.1 µV/24 h, 3.6 Ω, 2.993 nV/√Hz@1 Hz, and less than 70 µV/°C, respectively. Additionally, the AgNP-CF electric field sensor demonstrates low polarization and high stability. In field and simulated ocean tests, the AgNP-CF electrode exhibits excellent performance in the field and underwater environments, which renders it promising for the measurement of the ocean and geoelectric fields owing to its advantages, such as low noise and high stability.

## 1. Introduction

The geodetic electricity method and ocean electricity method map subsurface conductivity distributions by measuring variational natural electric fields, making them essential tools for geological prospecting, resource evaluation, disaster mitigation, ecological monitoring, and climate research applications across various scientific and industrial fields [1,2,3]. Telluric and marine electric field measurements typically employ two main electrode types: metallic electrodes, including copper (Cu) rods and stainless steel rods, and nonpolarizing electrodes, such as silver/silver chloride (Ag/AgCl), lead/lead chloride (Pb/PbCl), and inert electrodes [4,5,6,7]. Metallic electrodes offer cost advantages but suffer from polarization effects that distort self-potential measurements, leading to their gradual replacement by nonpolarizing electrodes [8,9]. Metal compound electrodes such as Ag/AgCl operate through redox reactions following the Nernst equation at the electrode interface [10]. At the electrochemical equilibrium between the anode and cathode redox reactions, the potential difference of the electrode pair theoretically reaches zero. In this state, the self-potential effect on measurement accuracy becomes negligible. Such electrodes exhibit superior performance in terms of self-potential, high stability, and minimal self-noise, making them ideal for precision electric field sensing applications. However, Ag/AgCl electrodes require stable redox reactions and consistent electrolyte conditions to maintain their self-potential stability; therefore, they are particularly sensitive to environmental variations [11]. Moreover, Ag/AgCl electrodes gradually consume their active materials during redox reactions, increasing maintenance costs and reducing service life [12]. Consequently, current research focuses on extending the electrode lifespan and reducing maintenance costs while preserving high performance [13,14]. In addition to improving metal compound electrodes, developing novel electrode materials is another key research focus [15].

Meanwhile, carbon is a chemically inert material. Therefore, electrodes based on carbon fiber have high electrochemical stability [16]. This stable electrode merely serves as a passive current conductor and hardly participates in REDOX reactions, demonstrating minimal sensitivity to environmental ion concentrations, which renders it an exceptional sensor material [17]. However, unmodified CF electrodes develop electrical double-layer capacitance (EDLC) during operation. Because no redox reactions occur, charge transfer relies solely on physical adsorption–desorption processes within EDLC. This mechanism, which is limited by ion migration rates, cannot track rapid high-frequency signal variations, resulting in measurable response lag and signal attenuation during dynamic measurements [18]. Additionally, the inherent electrochemical inertness decreases conductivity compared with traditional metal electrodes, requiring surface modifications to enhance the conductivity and high-frequency response characteristics. Research has confirmed that tailored surface treatments can substantially improve these performance parameters [16].

J. Yin et al. wrapped slender Ag nanowires around the surface of exposed carbon nanotubes, considerably increasing the active area and electron-transfer rate of these carbon nanotubes [19]. X. M. Zhang et al. notably enhanced the conductivity of flexible conductors by combining Ag nanoparticles (AgNPs) with CFs [20]. In the context of CF modification, improving interfacial strength between a modified material and CFs is a key research point. J. Yang et al. designed a silk-like coating on the CF surface, closely combining a bundled Ag coating and CFs. The strength and toughness of the composite material increased by 87.1% and 70.2%, respectively, and electrical conductivity reached 2.4 × 10^−5^ S/M [21]. C. Wang et al. successfully fabricated a novel double-layer reinforced CF composite, integrating AgNPs and graphene oxide (GO) into CFs through electrochemical deposition and electrophoretic deposition and thus considerably improving the surface roughness and mechanical strength of CFs [22].

Surface modification can further enhance the corrosion resistance and environmental stability of CF electrodes. C. Fu et al. found that Ag and CF composites could enhance the arc erosion resistance of materials [14]. Moreover, a reduced GO (rGO)-CF van der Waals (vdWs) heterostructure prepared by B. Zhan et al. exhibited multiple functions, such as good corrosion resistance and excellent thermal insulation performance [23]. CF electrodes developed by Polyamp AB had electrical conductivity, corrosion resistance, and antipolarization characteristics and were often used in underwater sensors to detect weak electric fields. Their CF product together with their differential amplifier PA3004 could detect weak electric fields down to 1 nV/m with frequency range 10 mHz to 5000 Hz [24].

Z. Hu et al. fabricated a novel Ag-modified CF electrode with a range drift of 29 μV/24 h, a relatively low AC impedance of 1.42 Ω@1 Hz, and an electrochemical self-noise of 1.38 nV/rt (Hz)@1 Hz [17]. Z. Hu et al. prepared a dual-carbon substrate Ag–rGO–CF composite electrode with rGO as the load using a simple closed oxidation and hydrothermal method. The prepared Ag–rGO–CF composite electrode was characterized by a honeycomb-like rGO conductive framework and dispersed AgNPs and exhibited excellent electrode properties, such as low resistance, high electrochemical reaction rate, stable potential, long-term stability, and low cost [6]. These studies provided support for the application of modified CF electrodes in the measurement of the electric fields of earth and the ocean; however, research in related applications is still in its infancy.

In order to solve the problems such as poor wettability and high interface impedance of traditional carbon fibers, which result in poor frequency response and large potential drift, this paper describes the design and preparation of a dendritic AgNP-modified CF (AgNP-CF) electrode using a combination of roughening pretreatment and electroplating. Specifically, the roughening pretreatment consisting of thermal oxidation and electrochemical oxidation resulted in a rough CF surface full of grooves, and subsequent modification of the CFs with AgNPs via electroplating furnished the AgNP-CF electrode. The electrode was subjected to microscopic characterization and self-potential, self-noise, temperature drift, and signal response tests. Moreover, an AgNP-CF sensor circuit system was applied to field electrical exploration experiments and laboratory underwater environment tests. The particle size distribution of the modified material was 400–600 nm, the average impedance of the electrode was 3.6 Ω, the range drift was 52.1 μV/24 h, the self-noise was 2.993 nV/√Hz@1 Hz, the temperature drift was less than 70 µV/°C, and the signal response error rate was less than 0.38%. The experimental results show that the AgNP-CF electrode has the advantages of a long service life, maintenance-free operation, low impedance, high stability, and high sensitivity, providing technical support for the measurement of electric field signals in long-term and complex environments.

## 2. Theoretical Analysis

### 2.1. Electrode Polarization

During electric field measurements, electrode polarization results from multiple physical and chemical mechanisms, among which electrochemical polarization is the dominant factor.

When metallic electrodes contact the electrolytes, redox reactions at the electrode surface cause localized charge accumulation. This shifts the electrode potential from its equilibrium state, creating polarization potentials that distort measurement accuracy. In contrast, metal compound electrodes undergo reversible reactions with stable reactants. For example, the Ag/AgCl electrode undergoes the following reversible reaction:

(1) $$Ag+Cl^{-}\to AgCl+e^{-}$$

This reversibility maintains stable electrode potentials without charge accumulation, considerably reducing polarization effects.

CF electrodes demonstrate a fundamentally different behavior. As chemically inert materials, they undergo negligible redox reactions in most environments, resulting in minimal polarization. This inherent stability makes CFs an ideal nonpolarizing electrode material.

### 2.2. Surface Modification of CFs

CF electrodes exhibit exceptional stability and minimal polarization effects, remaining electrochemically inert during measurements, with charge transfer occurring primarily via EDLC charging–discharging. For high-frequency signal acquisition, rapid interfacial charge–discharge cycles must track electric field variations. The EDLC time constant is given by:

(2) $$\tau =R_{ct}\cdot C_{dl}$$

where *R_ct_* is the charge transfer resistance and *C_dl_* is the double-layer capacitance. The high specific surface area of the CFs results in substantially large *C_dl_* values. In the high-frequency range, the interfacial impedance of the CF electrodes is predominantly governed by the double-layer capacitance, with a considerably increased *C_dl_* decreasing the signal transmission efficiency.

Surface roughening pretreatment and modification techniques effectively enhance the surface roughness and conductivity of CFs. This improves their chemical activity and wettability, thereby reducing interfacial capacitance while accelerating charge transfer rates, ultimately improving signal response capabilities.

The naturally smooth surface of CFs exhibits poor combination with modifier particles. Therefore, a pretreatment involving surface roughening is essential to modify the surface morphology of CFs. During roughening, the graphite-like structure on the CF surfaces becomes disrupted, forming micron- and nanoscale grooves that increase surface roughness. This enhanced topography promotes mechanical interlocking between the CFs and modifiers, strengthening interfacial bonding performance.

The modification of AgNPs dramatically increases the effective surface area of CFs, exposing more electrochemically active sites [18]. This enhances ion-exchange capacity between the electrode and electrolyte solution, improving sensor sensitivity and detection limits. Simultaneously, AgNPs form high-density conductive networks across the CF surface [25], reducing electron transport path resistance and accelerating charge transfer rates and thereby collectively boosting the signal response of the electrode [26].

## 3. Preparation and Characterization

### 3.1. Materials and Methods

In order to roughen the surface of carbon fibers and enhance the bonding strength between the modified particles and the carbon fibers, the carbon fibers were treated by a combination of electrochemical oxidation and thermal oxidation. First, electrochemical oxidation of carbon fibers was carried out in an alkaline environment. The main electrochemical reactions that occurred were as follows [26,27]:

(3) $${\mathrm{OH}}^{-}\to \cdot \mathrm{OH}+e^{-}$$

(4) $$C+4\cdot \mathrm{OH}\to CO_{2}↑+2H_{2}O$$

As shown in (3), *OH^−^* is oxidized to hydroxyl radicals (*·OH*) at the anode. Subsequently, as shown in (4), in an alkaline environment, hydroxyl radicals(*·OH*) oxidize the surface of carbon fibers, generating *CO*_2_ gas and *H*_2_*O*, forming a striped groove structure on the surface of the carbon fibers, thereby increasing the specific surface area of the carbon fibers.

Then, the thermal oxidation of carbon fibers was carried out. The reactions involved in etching during the thermal oxidation process were as follows:

(5) $$C+O\to CO_{2}↑$$

(6) $$2C+O_{2}\to 2C=O$$

(7) $${C+H}_{2}O\to -\mathrm{COOH}$$

As shown in (5), oxygen (*O*_2_) gas diffuses and combines with the active sites on the surface of carbon fibers, reacting to generate carbon dioxide (*CO*_2_) gas. This is the main cause of thermal oxidation etching. Meanwhile, as shown in (6) and (7), *C* also combines with oxygen to form a carbonyl group (*C=O*) on the surface of carbon fiber, and a small amount of water vapor reacts with carbon (*C*) to form a carboxyl group (*-COOH*). Several reactions act together to form a network structure with interwoven grooves on the surface of carbon fibers, further increasing the specific surface area.

AgNPs were modified on the surface of carbon fibers by electroplating, and a silver nitrate solution was used as the electrolyte. The anode was carbon fiber, and the platinum (Pt) electrode served as the cathode. Reactions occurred respectively at the cathode and anode:

(8) $${\mathrm{Ag}+e}^{-}\to \mathrm{Ag}\;(\mathrm{Cathode})$$

(9) $$2H_{2}O\to O_{2}+4H^{+}+4e^{-}\;(\mathrm{Anode})$$

As shown in (8), under the action of voltage, silver ions (*Ag^+^*) gradually diffused to the surface of the cathode carbon fiber and combined with electrons e^−^ to form silver atoms. The silver atoms grew into nanoparticles and aggregated into clusters in the grooves on the surface of the carbon fiber, forming AgNPS-modified CFs.

Graphene oxide (*GO*) was modified on the surface of carbon fibers by electrophoretic deposition [28]. Voltage was applied to the graphene oxide dispersion, with carbon fibers as the anode and Pt electrodes as the cathode [29,30]. The following reactions occurred during the electrophoretic deposition process:

(10) $$2H_{2}O+2e^{-}\to \;H_{2}↑+2OH^{-}(Cathode)$$

(11) $$GO-(\mathrm{Disperse})\to GO(\mathrm{Deposition})+e^{-}(Anode)$$

The surface of graphene oxide contains a large number of carboxyl groups, hydroxyl groups, and epoxy groups (*-C-O-C-*), which carry a strong negative charge when ionized in water. Under the action of voltage, dispersed graphene oxide (*GO-*) aggregates and deposits in the grooves on the surface of the anode carbon fiber, forming Go-modified CFs [7].

During the thermal reduction process, the deoxidation reaction mainly occurred. Under high-temperature conditions, various oxygen-containing groups in graphene oxide were separated in the form of gaseous products (*CO*_2_, *H*_2_*O*, *CO*) and restored into *C-C* bonds. rGO-modified CFs electrodes were formed. At this point, the conductivity of the electrode was significantly improved.

### 3.2. Roughening Pretreatment

**Figure 1:**
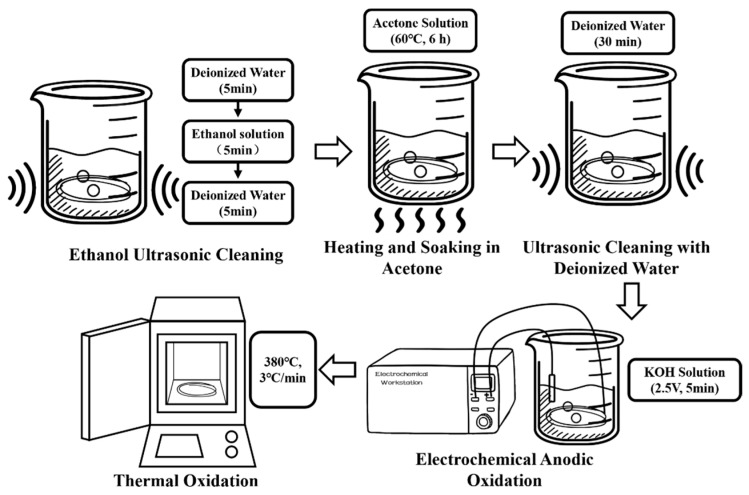
Schematic of pretreatment.

The surface roughening pretreatment of CFs was performed using a combined method of electrochemical oxidation and thermal oxidation. The preparation process is shown in Figure 1.

CF bundles (24 K, 10 cm) were immersed in deionized water and cleaned in an ultrasonic cleaner for 5 min. Subsequently, the CF bundles were soaked in a 75% ethanol solution prepared using deionized water and anhydrous ethanol and ultrasonically cleaned for 5 min. Finally, the CF bundles were immersed again in deionized water and ultrasonically cleaned for 5 min to dissolve oil stains and release agents or organic impurities such as paint and resin residues attached to the CF surface without damaging the chemical bonds of CFs or reducing their performance.

The CF bundles were immersed in a beaker containing a 50 wt% acetone solution, sealed with parafilm to prevent acetone volatilization, placed on a magnetic stirrer, and heated at 60 °C for 6 h to clean the epoxy coating on the CFs surface. The CF bundles were then immersed in deionized water and ultrasonically cleaned for 30 min until a neutral pH was reached. The anode of the electrochemical workstation was connected to a CF bundle, and the cathode was connected to a platinum (Pt) electrode for electrochemical anodic oxidation. A 5 wt% KOH solution was used as the electrolyte. The oxidation voltage and duration were 2.5 V and 5 min, respectively. The cleaned CF bundles were placed in a muffle furnace and subjected to thermal oxidation treatment at 380 °C with a heating rate of 3 °C/min and then cooled to room temperature.

The surface morphology of CFs before and after pretreatment is shown in Figure 5a, where obvious grooves can be observed on the pretreated CF surface due to etching.

### 3.3. Surface Modification

#### 3.3.1. Modification with AgNPs

**Figure 2:**
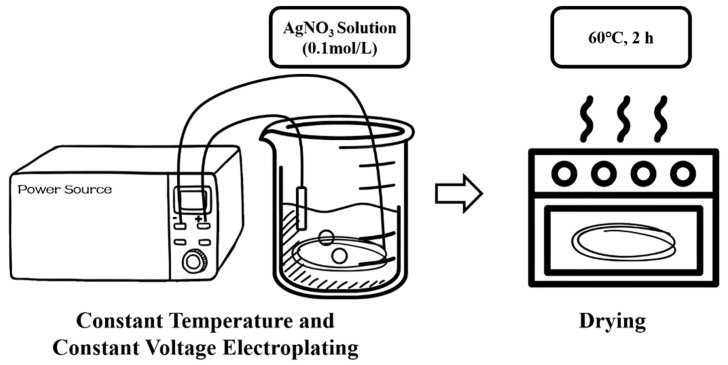
Electroplating and drying.

As shown in Figure 2, the AgNP-CF electrode core was prepared using a constant-temperature and constant-voltage electroplating method in a water bath. Electroplating deposits AgNPs onto the substrate surface through cathodic reduction reactions, thereby enhancing conductivity and reducing interface capacitance [31].

After roughening, the CF bundles were cut into short strips with an approximate length of 10 cm long and width of 1 cm. A Pt electrode and CF bundles were connected to the anode and cathode of a constant-voltage power supply, respectively, and immersed in a 0.1 mol/L AgNO_3_ solution. Constant-temperature electroplating was performed in a water bath while manually adjusting the Pt electrode position to ensure a uniform modification.

The electroplating rate during the electroplating process is an important parameter affecting the electroplating effect, and the factors influencing the electroplating rate include voltage, temperature, and the concentration of the electrolyte solution. The higher the voltage is, the faster the electroplating rate will be. The higher the temperature, the faster the electroplating rate. Under the condition that the concentration of the electrolyte solution remains unchanged, the optimal electroplating parameters are sought by changing the electroplating voltage and temperature. The AgNP-CF electrodes were prepared under different reaction conditions: 4 V and 20 °C, 4 V and 50 °C, 4 V and 80 °C, and 8 V and 50 °C. After 10 min of electroplating and heating at 60 °C in a drying oven for 2 h, AgNP-modified CF (AgNP-CF) electrodes were obtained.

#### 3.3.2. Modification with rGO

**Figure 3:**
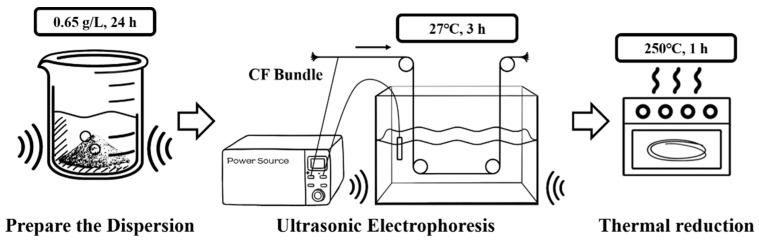
Electrophoresis and thermal reduction.

For comparison, CF electrodes modified with rGO (rGO-CFs) were prepared using an electrophoretic deposition method, as shown in Figure 3.

**Figure 4:**
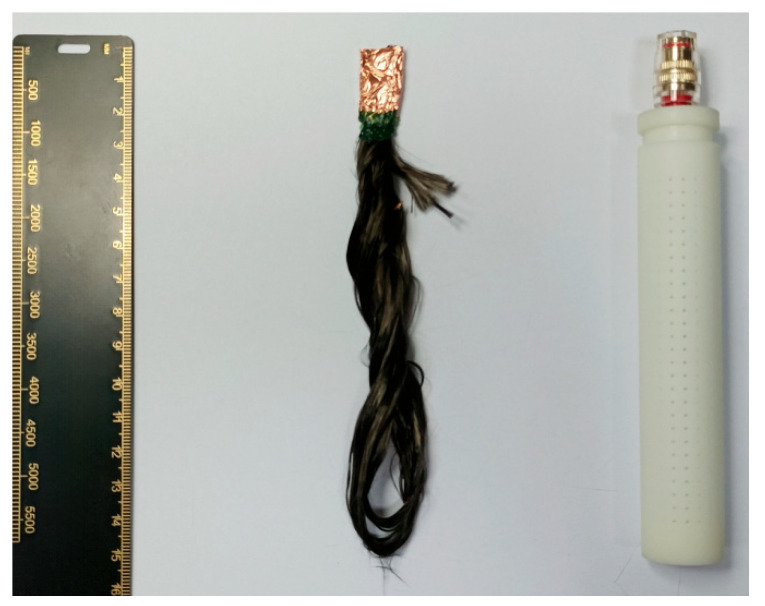
Encapsulated electrode.

The rGO-CF electrode was prepared as follows: A 0.65 g/L GO suspension was prepared by mixing appropriate amounts of GO with deionized water, followed by ultrasonication for 24 h to evenly disperse the GO in deionized water. Owing to the presence of carboxyl and hydroxyl groups, GO carries negative charges in an aqueous solution and migrates toward the anode under an electric field. Using GO dispersion as the electrolyte, CF bundles were connected to the anode and a Pt electrode to the cathode. Electrophoretic deposition was conducted for 3 h at 27 V. The deposited electrodes were then reduced in a drying oven at 250 °C for 1 h before encapsulation to obtain the rGO–CF electrodes. The encapsulated electrode is shown in Figure 4. One end of the electrode was connected to the joint through a wire, and the connected part was sealed with epoxy resin to prevent corrosion. The packaging shell adopted a double-layer porous structure. Under the premise of ensuring the ion exchange capacity inside and outside, it prevented the electrode core from being damaged by the environment [8].

### 3.4. Characterization

The surface morphology of the AgNP-CF electrodes was observed using optical microscopy and scanning electron microscopy (SEM) to analyze the modification of AgNPs under different temperature and voltage conditions.

**Figure 5:**
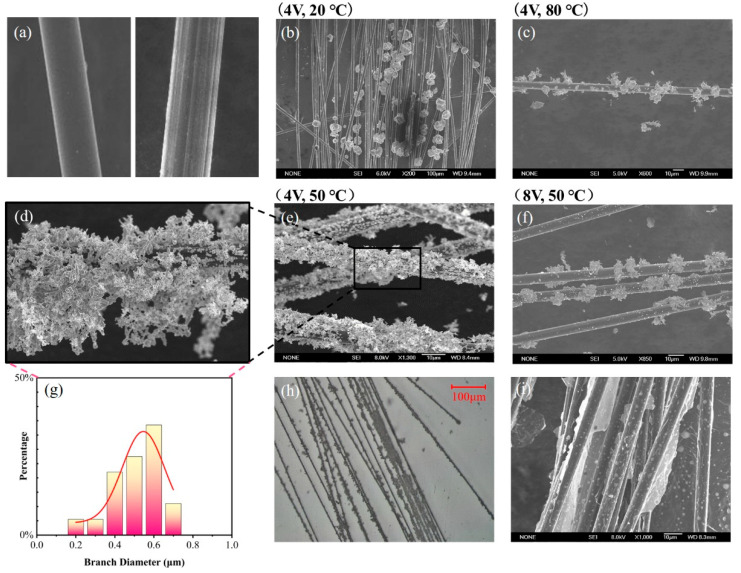
Electrode characterization. SEM images of (**a**) carbon fibers (CFs) before and after pretreatment and AgNP-CFs prepared at (**b**) 4 V and 20 °C and (**c**) 4 V and 80 °C. (**d**) Partial enlarged view of (**e**). (**e**) AgNP-CFs prepared at 4 V and 50 °C and (**f**) 4 V and 80 °C. (**g**) Branch diameter distribution map of AgNP-CFs prepared at 4 V and 50 °C. (**h**) Optical microscopy image of AgNP-CFs prepared at 4 V and 50 °C. (**i**) SEM image of rGO-CFs.

Optical microscope images of the AgNP-CF electrodes are shown in Figure 5. As shown in Figure 5c,f, the AgNP-CF electrodes prepared under the conditions of 8 V and 50 °C and 4 V and 80 °C displayed a relatively low degree of modification and were surrounded by small Ag crystal particles remaining after Ag crystal peeling. This can be attributed to an increase in current density as temperature or voltage increased, rapidly decreasing Ag ions on the cathode surface, causing Ag crystals to grow and weaken bonding force with the substrate, resulting in the peeling-off of Ag crystals.

As shown in Figure 5b, Ag crystals on the surface of the AgNP-CF electrode prepared under the conditions of 4 V and 20 °C were coarse and spherical. The low modification temperature led to a reduction of the ion diffusion rate and the cathode reaction rate, decreasing the Ag deposition rate, prolonging the modification time, making it susceptible to incomplete modification, weakening the cathode polarization effect, and slowing down the formation speed of Ag crystal nuclei.

As shown in Figure 5d, Ag crystals on the surface of the AgNP-CF electrode prepared under the conditions of 4 V and 50 °C were dendritic, uniformly modified, and had a relatively high specific surface area.

The size distribution of Ag crystals is shown in Figure 5g. Branches with a diameter of <1 μm were mainly distributed between 400 and 600 nm, effectively increasing the specific surface area of the AgNP-CF electric field sensor.

**Figure 6:**
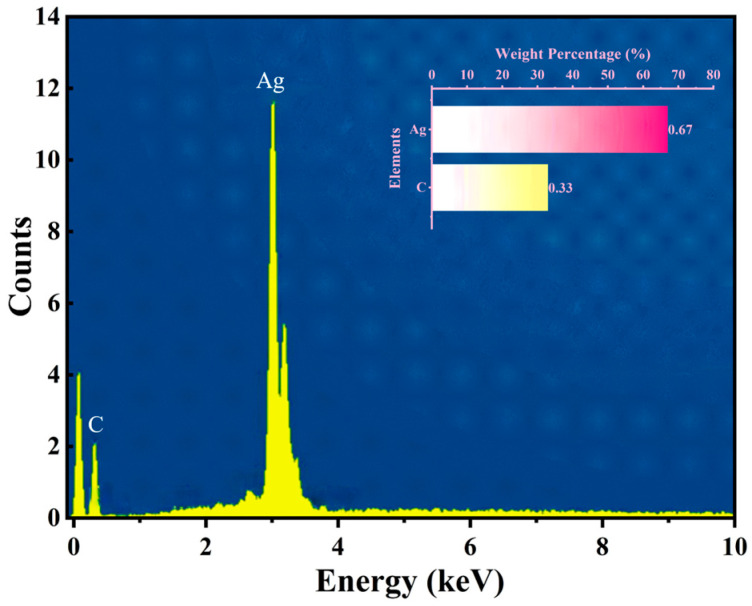
EDS analysis of AgNP-CFs prepared at 4 V and 50 °C.

As shown in Figure 6, the energy-dispersive spectroscopy (EDS) analysis of the AgNP-CF electrodes indicated C and Ag contents of 33.0% and 67.0%, respectively, confirming successful surface modification without impurities.

As shown in Figure 5i, the SEM images of the rGO-CF electrodes reveal complete surface modification, with rGO forming film-like and sheet-like structures on the CF surface, substantially increasing the specific surface area of the rGO-CF electrode.

**Figure 7:**
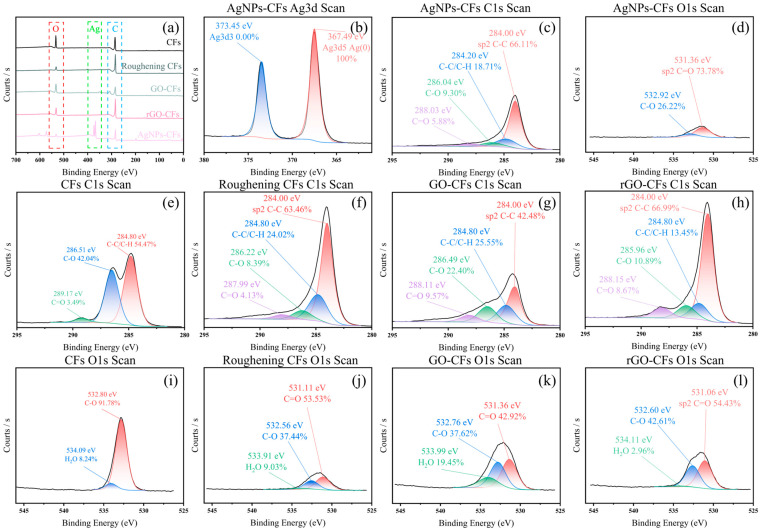
XPS test results. (**a**) XPS full spectra of CFs, roughening CFs, GO-CFs, rGO-CFs, and AgNP-CFs. (**b**) Ag3d splitting spectrum of AgNP-CFs. (**c**) C1 splitting spectrum of AgNP-CFs. (**d**) O1 splitting spectrum of AgNP-CFs. (**e**–**h**) The C1 segregation spectra of CFs, roughening CFs, GO-CFs, and rGO-CFs. (**i**–**l**) The O1 segregation spectra of CFs, roughening CFs, GO-CFs, and rGO-CFs.

The chemical compositions of CFs, roughening CF, AgNP-CFs, GO-CFs, and rGO-CFs were analyzed by XPS, and the results are shown in the Figure 7. As shown in Figure 7a, the content of *O* element in the carbon fiber is significantly reduced after roughening, existing in the form of gaseous *CO_2_* and *H_2_O*, leaving striform grooves on the surface of the carbon fiber. Meanwhile, as shown in Figure 7j, *C* combined with *O*_2_ to form *C=O*, which is consistent with (6). After modification with AgNPs and drying, the grooves on the surface of carbon fibers were occupied by AgNPs, and *H*_2_*O* volatilized in the form of gas. Compared with roughening CFs, the changes in *C* and *O* elements were not significant.

After modification with graphene oxide, the content of the *O* element in Go-CFs increased, and as shown in Figure 7k. The *O* element mainly exists in the form of *C=O* and *C-O*. After Go was reduced to rGO, some of the *O* elements volatilized in the form of gaseous H_2_O and CO_2_, and some *O* elements combined with *C* elements to form *C=O*, resulting in a decrease in the content of *O* elements. Some *C-H* and *C-O* decomposed to form *C-C*, resulting in a decrease of *C-H* and *C-O* and an increase in *C-C*. Due to the reasons of material preservation and air humidity, there was still a small amount of *H*_2_*O* in rGO-CFs.

The XPS test results indicate that AgNPs were successfully modified on the surface of CFs by electroplating, and rGO by electrophoresis was also modified on the surface of CFs, which is in line with the theoretical analysis.

## 4. Test and Results

### 4.1. Self-Resistance

**Figure 8:**
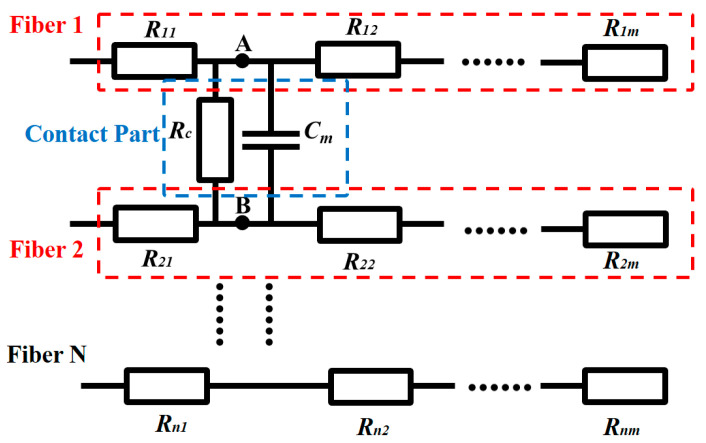
Equivalent impedance model diagram of theAgNP-CF electrode.

The equivalent impedance model of the AgNP-CF electrode is shown in Figure 8 [32], where the impedance of a single fiber is regarded as a series resistance unit, and there are contact resistance (*R_c_*) and contact capacitance (*C_m_*) between the fibers. Ideally, the resistance of a single fiber is uniformly distributed, and all the resistance units exhibit the same resistance value *R_f_*. When the current passes through, the voltages at node A and node B are the same, and the voltage between them is 0. At this time, the impedance of the unit can be expressed as:

(12) $${R=(R}_{11}{+R}_{12}{)//(R}_{21}{+R}_{22}{)=R}_{f}$$

The magnitude of *R_f_* is related to the content of the CF conductive medium and surface-modified AgNPs. Therefore, the impedance of the CF electrode and the AgNPs-CF electrode mainly depends on their own resistance.

The resistance of the CF electrode and AgNP-CF electrode in an electromagnetic shielded environment at normal temperature was directly measured using a DMM6500 digital multimeter (Keithley of Tektronix, Cleveland, OH, USA) in the dual-wire resistance mode, which directly read the results. The CF electrode and AgNP-CF electrode contained the same number of fibers. The sample size of the test was five.

The test results are shown in Table 1. The average self-resistance of the CF electrode was 29.2 Ω and that of the AgNP-CF electrode was 3.6 Ω. This demonstrates that the dendritic AgNP-CF electrode developed in this study using the roughing pretreatment modification combined with constant-temperature and constant-voltage electroplating had a relatively low source resistance, meeting the requirement of low impedance for electric field sensors.

### 4.2. Potential Drift

Electrode potential drift refers to the difference between the maximum and minimum output potentials of an electric field sensor over a certain period, reflecting the fluctuation range of the output signal of the sensor during long-term use. Therefore, this parameter serves as a crucial indicator of sensor stability.

**Figure 9:**
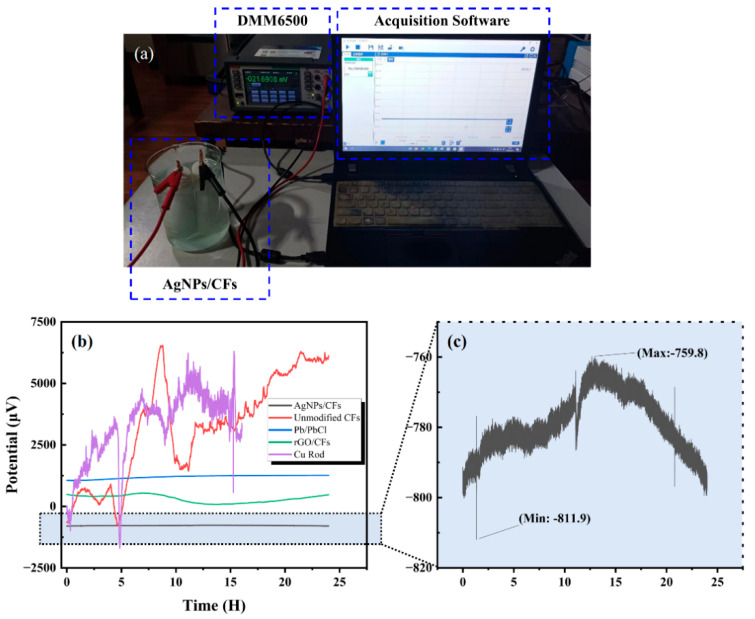
Potential drift test. (**a**) Experimental setup for potential drift test. Self-potential changes measured for 24 h for (**b**) the five types of electrodes and (**c**) AgNP-CF electrode.

As shown in Figure 9a, the sensor was placed in a 3.5% NaCl solution within an electromagnetic shielding environment. After the electrodes reached a stable state (with current density, reaction rate, surface temperature, and other parameters being stabilized), the potential variation in the electrode pair was measured using a DMM6500 6½-digit multimeter (Keithley of Tektronix, Cleveland, OH, USA). For comparison, the self-potentials of rGO-CFs, unmodified CFs, Pb/PbCl, and Cu rod electrodes were also measured for 24 h.

As shown in Figure 9b,c, the results indicate that the AgNP-CF electric field sensor exhibited a potential drift of 51.2 μV/24 h, demonstrating high stability and meeting the requirements for marine electric field detection and terrestrial electrical prospecting. Meanwhile, the rGO–-electric field sensor showed a considerably higher drift of 461.1 μV/24 h. Compared with the unmodified carbon fiber electrode, the rGO-CF electrode, due to the highly conductive network constructed by the rGO modification layer, reduced the interface impedance on the electrode surface and improved the electron migration efficiency, making the rGO-CF electrode have higher stability than the unmodified carbon fiber electrode. However, compared with AgNPs, the conductivity of rGO is weaker, and rGO physically adheres to the surface of carbon fibers and is prone to fall off, resulting in the stability of the rGO-CF electrode being weaker than that of the AgNP-CF electrode. therefore, the rGO-CF electrode was excluded from further testing. Table 2 summarizes the specific potential drift values of the five electrode pairs.

### 4.3. Self-Noise

The self-noise of an electric field sensor refers to random electrical signals generated by the sensor owing to the presence of internal materials or environmental factors in the absence of an external electric field input. This type of noise will affect the weak electric field signal response of the sensor, reducing measurement accuracy. The value obtained from sensor noise measurement is the sum of self-noise generated by the electrode and system noise produced by the measurement system, that is, total voltage noise. Therefore, when calculating sensor noise, the noise of the measurement system needs to be subtracted from the measurement result to improve the accuracy of the measurement data. The total voltage noise and voltage noise of the test system were determined as follows. The electrode pair was connected to a multifunctional digital multimeter DMM6500 (at a range of 100 mv, the resolution is 100 nV) in the DC voltage measurement mode with a sampling NPLC of 0.01 and a measurement time of 8,000,000 points to obtain the total voltage noise (*v_1_(t)*). When the multifunctional digital multimeter DMM6500 was short-circuited, the test parameters remained unchanged. The voltage noise of the test system (*v_2_(t)*) was determined.

Noise power spectral density describes the power distribution of noise signals at different frequencies and is usually used to analyze the characteristics of random signals in the frequency domain. The fast Fourier transform (FFT) of the total voltage noise (*v_1_(t)*) was performed to obtain the spectral function (*V(f)*) of voltage noise.

(13) $$V(f)=FFT(v_{1}(t))$$

The power spectral density (*P*(*f*)) is:

(14) $$P(f)={\left|V(f)\right|}^{2}$$

By substituting *P*(*f*) and the frequency resolution (*df*) into the following formula, the amplitude spectral density (*S_v_*(*f*)) of the combined electrode self-noise and the measurement system noise, which is denoted as *N* (V/√Hz @1 Hz), is calculated in units of V/√Hz@1 Hz:

(15) $$S_{v}(f)=\sqrt{\frac{P(f)}{df}}$$

Similarly, the system voltage noise at 1 Hz is obtained as *N*_2_ (V/√Hz @1 Hz).

Finally, the self-noise of the AgNP-CF electrode was calculated. The total noise refers to the total noise of the electrode and the test system. Electrochemical noise originates from random changes in voltage or current and belongs to the category of random noise. Its magnitude is usually expressed as the mean of the squares of the variables. There is no correlation between the voltage noise of the test system and the background noise of the electrodes. The self-noise of the electrode at 1 Hz (*N*_1_) can be expressed as:

(16) $$N_{1}=\sqrt{N^{2}-{N_{2}}^{2}}$$

**Figure 10:**
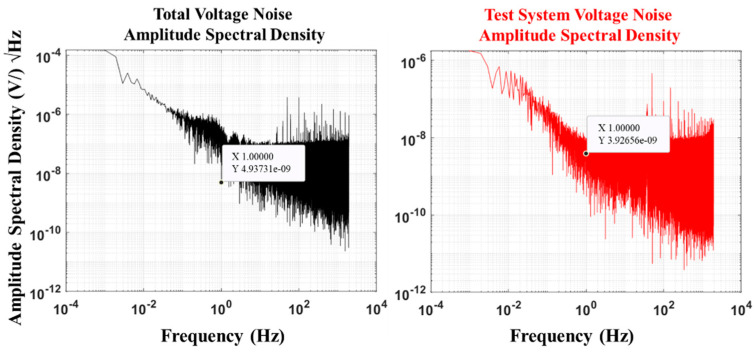
Self-noise test results.

The total voltage noise of the AgNP-CF electrode and the voltage noise test results of the test system are shown in Figure 10. The self-noise test result of the AgNP-CF electrode was calculated to be:

(17) $$\sqrt{4.937^{2}-3.926^{2}}=2.993\mathrm{nV}/\sqrt{\mathrm{Hz}}@1\;\mathrm{Hz}$$

In addition, we tested the self-noise of the unmodified carbon fiber electrode and compared it with that of the rGO-CF electrode. The test results are shown in Table 3. These results show that the AgNP-CF electrode has a relatively low self-noise.

### 4.4. Temperature Drift

**Figure 11:**
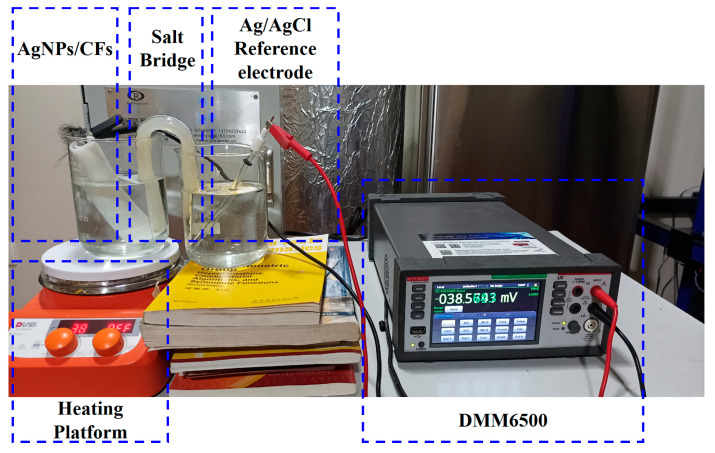
Experimental setup for the temperature drift test.

Temperature drift is an amplitude change in the output signal of the sensor due to temperature variations and is usually expressed in V/°C or as a percentage. For electrode pairs, it reflects the fluctuation range of the differential output signals of the two electrodes during changes in the external environmental temperature and is an important parameter for evaluating sensor stability. As shown in Figure 11, during the test, the No. 1 electrode of the AgNP-CF electrode pair was placed in the No. 1 beaker (3.5% NaCl solution), which was then placed on the heating stage, and the Ag/AgCl reference electrode was placed in the No. 2 beaker (3.5% NaCl solution). The two beakers were connected by a salt bridge (U-shaped, spacing 35 mm, 15 × 150 mm, inside: potassium chloride solution and agar), and heating was started when the electrode reached a stable state. Meanwhile, an RC-4 thermometer was used to record temperature every 10 s. Measurement was conducted in an electromagnetic shielded environment using a DMM6500 multimeter (Keithley of Tektronix, Cleveland, OH, USA) to record potential at a sampling frequency of 0.1 Hz. After the test of electrode No. 1 in the electrode pair was completed, it was replaced with electrode No. 2, and the above experiment was repeated.

The calculation formula for temperature drift obtained from the test is:

(18) $$V_{\mathrm{td}}=\frac{V_{\max}-V_{\min}}{T_{\max}-T_{\min}}$$

Here, V_td_ represents the temperature drift of voltage with the unit of V/°C, *V_max_* and *V_min_* represent the maximum and minimum values of voltage or potential difference, and *T_max_* and *T_min_* represent the maximum and minimum values of temperature.

**Figure 12:**
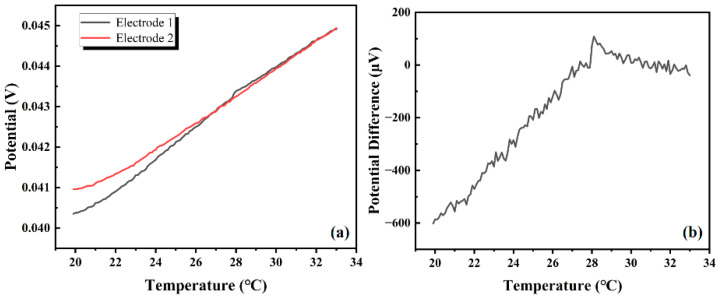
Temperature drift test results. (**a**) Potential variation with temperature and (**b**) potential difference variation with temperature of an AgNP-CF electrode pair.

As shown in Figure 12a, the temperature drift test results indicate that the temperature drift of the electrode No. 1 was 285 µV/°C and that of the electrode No. 2 was 328 μV/°C. The potential change trends of the two electrodes were extremely close.

As shown in Figure 12b, the change in the electrode potential difference was 66.03 µV/°C at 20–28 °C and 20.70 µV/°C at 28–33 °C. The results show that the temperature variation had almost no effect on the sensing performance of the AgNP-CF electrode pair, which exhibited good temperature stability.

### 4.5. Signal Response Test

**Figure 13:**
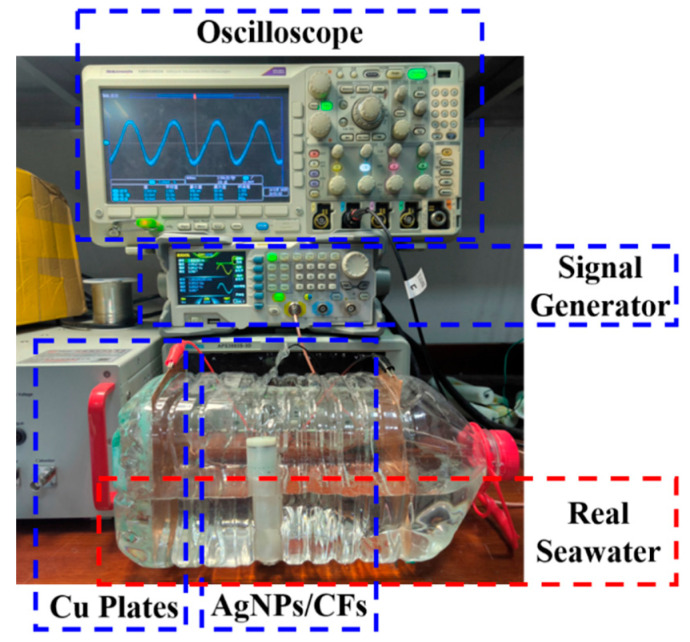
Experimental setup for real seawater signal response test.

**Figure 14:**
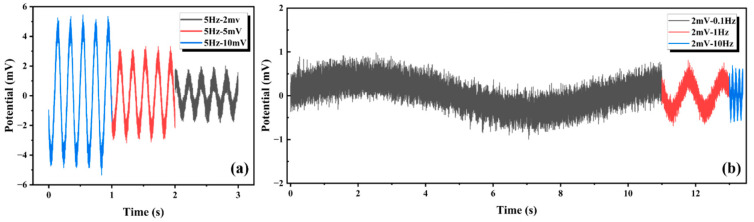
Results of signal response test in real seawater (PH = 7.9). (**a**) Signal response of the AgNP-CF electrode to an input signal with a frequency of 5 Hz and amplitudes of 2, 5, and 10 mV. (**b**) Signal response of the AgNP-CF electrode to an input signal with an amplitude of 2 mV and frequencies of 0.1, 1, and 5 Hz.

The experimental setup is shown in Figure 13, and the underwater electric field signal response was measured in real seawater. The AgNP-CF electrodes were immersed in the seawater solution (PH = 7.9) and connected to the oscilloscope, and flanked Cu plates were connected to the output of the signal generator. This configuration generated low-frequency weak signals simulating natural electric fields in seawater. The test results are shown in Figure 14.

The frequency error rate (*E_f_*) was calculated using the following formula:

(19) $$E_{f}=\frac{\left|\;f_{o}-f_{c}\;\right|}{f_{o}}\times 100\%$$

where *f_o_* is the output frequency of the signal generator and *f_c_* is the signal frequency collected by the oscilloscope.

As shown in Table 4, the maximum error was 0.36%, the minimum error was 0.13%, and the mean error was 0.22%. The AgNP-CF electrode maintained an excellent frequency fidelity, establishing an experimental foundation for the field application of AgNP-CF electrodes.

### 4.6. Application Test

A geoelectrical survey field test was conducted in Shaoguo Town, Changchun City, Jilin Province, China using a DZD-6B DC resistivity meter (China Geological Equipment Group Co., Ltd., China Instrument Co., Chongqing, China) with the four-electrode moving array method to measure apparent resistivity and apparent chargeability along the survey line. The survey line had a length of 100 m and measurement point intervals of 5, 10, 15, 20, 25, and 30 m. To verify the stability and low-frequency response capability of the AgNP-CF electrode in field conditions, natural potential and potential difference were measured at both ends of the survey line using four electrode arrays. The Cu rod electrodes and Pb/PbCl solid nonpolarizable electrodes were used as references.

**Figure 15:**
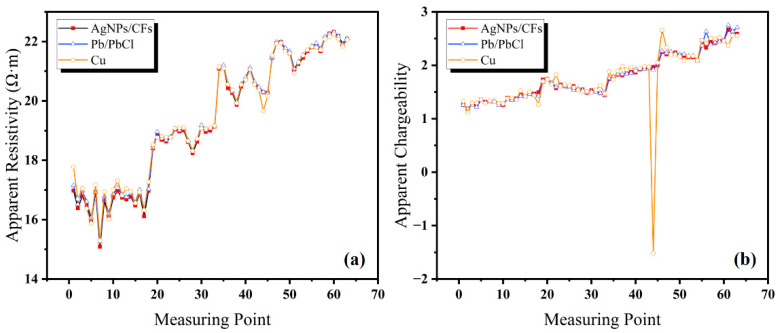
(**a**) Apparent resistivity (**b**) and apparent chargeability measured using the direct current method.

Sixty-three sets of data on apparent resistivity and apparent chargeability rate were collected for each electrode. As shown in Figure 15a, apparent resistivity showed minimal differences between the four electrode types, indicating consistent measurement performance.

As shown in Figure 15b, the apparent chargeability test results show that the AgNP-CF and Pb/PbCl electrodes produced highly consistent measurement values, while the Cu rod electrodes exhibited noticeable deviations.

All measurement sets for each electrode type showed some degree of received voltage error rate, reflecting measurement stability and accuracy. As shown in Table 5, the calculated error rates confirm that all four electrode types maintained low received voltage error rates, validating the reliability and accuracy of apparent resistivity and apparent chargeability measurements.

## 5. Conclusions

This research employed a combined thermal oxidation and electrochemical anodic oxidation roughing pretreatment process to modify CFs and prepare an AgNP-CF electric field sensor using constant-temperature and constant-voltage electroplating to address the issues of poor frequency response, large potential drift, and insufficient capacitance of CF electrodes. The surface morphology of the surface-modified CF electric field sensor was characterized using optical microscopy, SEM, and EDS. The main conclusions are as follows:

(1) Through optical microscopy, SEM experiments, XPS experiments, etc., it was proved that the AgNP-CF electrode surfaces were uniformly modified, substantially increasing the specific surface area.
(2) Using performance tests, the self-resistance, potential drift, self-noise, and temperature drift of the AgNP-CF electrode were determined to be 3.6 Ω, 52.4 μV/24 h, 2.993 nV/√Hz@1 Hz, and less than 70 µV/°C, respectively, demonstrating that the AgNP-CF electric field sensor possessed the advantages of low polarization and high stability. In the comparison with the CF electrode and the rGO-CF electrode, it is shown that AgNP-CFs have obvious advantages.
(3) Field tests of the electrical resistivity survey were conducted in Shaoguo Town, Changchun City, Jilin Province, along with simulated marine environment tests. The results indicated that the AgNP-CF electrode exhibited good performance in the field and underwater environments, providing technical support for measuring the ocean and geoelectric fields.

These conclusions all prove the good application prospects of the CF electric field sensor modified by AgNPs in geodetic electric field measurement and ocean electric field measurement. For comparison, the rGO-modified CF electric field sensor also had performance improvements compared to the unmodified CF electrode, but it did not reach the level of the AgNP-CF electric field sensor. Compared with Ag/AgCl electric field sensors, the modified carbon fiber electrodes had the characteristics of high stability, no maintenance, and low cost. However, AgNP-CF electrodes (<0.1 Ω, <10 μV/24 h, <1 nV/√Hz@1 Hz) have not been able to outperform Ag/AgCl electrodes in terms of source resistance, range drift, or self-noise. However, AgNP-CF electrodes (<0.1 Ω, <10 μV/24 h, <1 nV/√Hz@1 Hz) did not perform better than Ag/AgCl electrodes in terms of source resistance, range drift, or self-noise Further research and optimization are needed. Through the modification of different materials, the CF electrode can retain its own advantages such as high stability, long service life, and no maintenance while improving the wettability and conductivity of the electrode and reducing self-noise and source impedance. This research route has important research significance and application prospects for long-term and high-precision electric field measurement applications.

## Figures and Tables

**Table 1:** Self-resistance of the CF electrode and AgNP-CF electrode.

Measurement Number	Resistance of CFs (Ω)	Resistance of AgNP-CFs (Ω)
1	27.834	3.524
2	30.352	3.672
3	33.539	3.958
4	26.636	3.411
5	27.581	3.467
RSD (%)	9.57%	6.08%

**Table 2:** Comparison of the potential drift of different electrodes.

Types of Electrodes	Potential Drift (μV/24 h)
AgNP-CFs	52.1
rGO-CFs	461.1
Unmodified CFs	7562.6
Pb/PbCl	216.2
Cu Rod	7832.1

**Table 3:** Comparison of the self-noise of electrodes.

Types of Electrodes	Total Voltage Noise (nV/√Hz @1 Hz)	Test System Voltage Noise (nV/√Hz @1 Hz)	Self-Noise (nV/√Hz @1 Hz)
AgNP-CFs	4.937	3.926	2.993
rGO-CFs	339.1	3.926	339.0
Unmodified CFs	5723	3.926	5722

**Table 4:** Frequency error rate of signal response test.

*f_o_*	*f_c_*	*E_f_* (%)
5 Hz	4.991 Hz	0.18
5 Hz	5.018 Hz	0.36
5 Hz	5.015 Hz	0.33
0.1 Hz	99.85 mHz	0.15
1 Hz	998.1 mHz	0.19
10 Hz	9.987 Hz	0.13

**Table 5:** Error rate of different electrodes using the direct current method.

Electrodes Types	Max Error Rate (%)	Min Error Rate (%)	Average Error Rate (%)
AgNP–CF	0.5	0.0	0.12
Pb/PbCl	0.6	0.0	0.10
Cu	0.6	0.0	0.17

## Data Availability

The original contributions presented in this study are included in the article. Further inquiries can be directed to the corresponding authors.

## Abbreviations

The following abbreviations are used in this manuscript:

CF	Multidisciplinary Digital Publishing Institute
EDLC	Electrical double-layer capacitance
EDS	Energy-dispersive spectroscopy
FFT	Fast Fourier transform
GO	Graphene oxide
SEM	Scanning electron microscopy
